# The development of clinical thinking in trainee physicians: the educator perspective

**DOI:** 10.1186/s12909-020-02138-w

**Published:** 2020-07-16

**Authors:** Rachel Locke, Alice Mason, Colin Coles, Rosie-Marie Lusznat, Mike G. Masding

**Affiliations:** 1 University of Winchester, Winchester, UK; 2 University Hospital Southampton NHS Foundation Trust, Southampton, UK; 3 Health Education England Wessex, Winchester, UK

**Keywords:** Trainee physician, Clinical thinking, Medical education, Supervision, Medical educator

## Abstract

**Background:**

An important element of effective clinical practice is the way physicians think when they encounter a clinical situation, with a significant number of trainee physicians challenged by translating their learning into professional practice in the clinical setting. This research explores the perceptions of educators about how trainee physicians develop their clinical thinking in clinical settings. It considers what educators and their colleagues did to help, as well as the nature of the context in which they worked.

**Method:**

A qualitative approach was used in this study with in depth interviews carried out with educators as key informants. Rich data derived from 15 interview transcripts were analysed thematically in a rigorous and iterative process.

**Results:**

Three broad and overlapping themes were identified: working in an educationally minded culture; proximity of the educator to the trainee physician; and trajectory of the trainee physician. The departments in which these educators worked emphasised the importance for the education of trainee physicians. All members of the team were responsible for education of the team, and all members, particularly senior nurses, were able to give feedback upon the trainee physicians’ progress. Educators described working side by side with their trainee physician and frequently being in close proximity to them which means that the educator was both easily accessible and spent more time with their trainee physicians. They described a trajectory of the trainee physicians through the placement with close monitoring and informal assessment throughout.

**Conclusion:**

Recommendations are made as to how trainee physicians can be supported to develop their clinical thinking. Educators and managers can analyse their own and their department’s practice and select the recommendations relevant to their local circumstances in order to make change. This study adds the educator perspective to a body of literature about the importance of context and supportive learning environments. As such the discussion is applicable to the education of other health professionals.

## Background

An important element of effective clinical practice is the way physicians ‘think’ when they encounter a clinical situation. Different concepts have been used to describe the processes involved, including ‘clinical reasoning’ and ‘clinical problem-solving’, both of which suggest complex cognitive processes [1, 2]. Others suggest physicians largely engage in ‘problem definition’ rather than ‘problem-solving’ [3], which involves thinking about the problem, not necessarily solving it. Integral to this is allowing for doubt and uncertainty in clinical thinking [4].

Trainee physicians are supported in the development of their clinical thinking by their colleagues through supervision, and there is evidence that much learning results informally and unplanned from interactions with others in the workplace [5]. Good supervision includes overseeing training, modelling good practice, observing clinical encounters, and to engage in dialogue with the trainee physician [6]. Supervisors may even ask the person they are supervising about their thinking concerning a patient and compare it to their own [7].

**Fig. 1:**
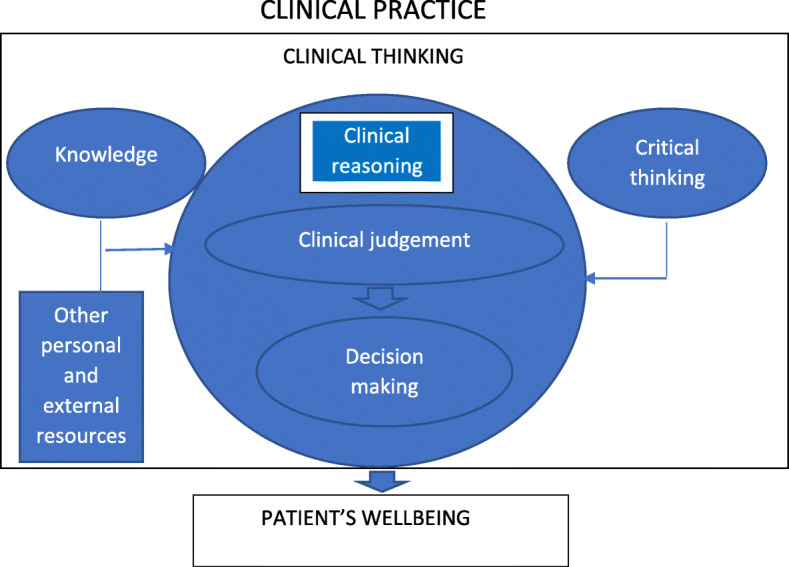
An Integrative Model of Clinical Thinking (Faucher, 2011)

From these perspectives, we believe ‘clinical thinking’ to be the most helpful term to use because it is a broader concept encompassing all these different cognitive processes [1, 8, 9]. See Fig. 1. One motive for undertaking the study presented here was that a significant number of trainee physicians referred to a Professional Support Unit at a UK postgraduate medical training organisation were described as finding it difficult ‘to think properly’ in the clinical setting.

The context in which this happens has been shown to be important. It is now more widely recognised that what was once termed competence is not a stable construct – that once acquired, competence would be retained always [10]. Rather, the capability of any professional practitioner to practise effectively and appropriately is greatly influenced by the context of their practice [5, 11, 12]. The suggestion is that a doctor may appear ‘competent’ in one context but not another – an observation also made by the Professional Support Unit mentioned above, which found that ‘failing’ trainees in one unit were found to ‘thrive’ in another.

There are indications that the context in which a doctor trains may determine the effectiveness of that training. A rigorous internal study of a UK postgraduate medical training organisation [13] showed that trainee physicians believed they learnt most in clinical units where they felt accepted and involved (a sense of “community”), were encouraged to contribute to the units’ work (a sense of “collegiality”), and could discuss their thoughts openly and honestly (a sense of “criticality”). These have been applied more recently in a study of the characteristics of effective training environments in obstetrics and gynaecology [14].

Our study aimed to explore the development of clinical thinking in trainee physicians with an emphasis on the importance of context rather than the individual’s cognitive processes [15]. Other research on context has sought to capture the medical trainees’ viewpoint in this regard [e.g. [16–18]. However, the focus of our work was on gaining the educator perspective since they, as noted above, are largely responsible for, and well placed to understand and comment on, the development of clinical thinking in trainee physicians.

The research explored the perceptions of educators about how trainee physicians developed their clinical thinking in clinical settings and what they, their colleagues, did, as well as the nature of the context in which they worked, to help develop clinical thinking of trainee physicians. The trainees referred to in this paper are FY2 doctors undertaking the Foundation Programme which is a two-year general postgraduate medical training programme in the UK which forms the bridge between medical school and specialist/general practice training. General recommendations are made as to how medical educators and their managers can support trainee physicians.

## Methods

### Research design

A qualitative approach was used in this study with in-depth interviews employed for data collection, as we have found this approach, rather than the collection of numerical data, enhances the potential more directly for change. Doing so enabled detailed narrative descriptions from the educators involved in the situations being researched and exploration of the complexity of connections in practice [19]. Fifteen semi-structured interviews were conducted with interviews lasting around 45 min each.

### Participants

Sampling was purposive to recruit participants who could provide rich and in depth information about the development of clinical thinking amongst trainee physicians. Medical clinicians were approached in departments with a local reputation for providing supportive learning environments as well as positive feedback from trainee physicians themselves, as reported in the General Medical Council (GMC) national training survey [20]. The educators in the sample also had a minimum of 3 year’s experience in their roles making them valuable ‘key informants’ [21]. They were all consultants, who had received training for their supervisory role as part of their post-training continuing professional development. In UK Foundation Training, all trainees have an educational supervisor (for their whole 2-year programme), and a clinical supervisor (for their time in the specific department) – our interviewees were all educational supervisors, clinical supervisors, or both.

### Ethical considerations

In carrying out the research the British Education Research Association’s (BERA) Ethical Guidelines for Educational Research were adhered to [22]. Informed written consent was gained from participants, including the right to confidentiality and to withdraw without explanation. The interviews were digitally recorded and once transcribed, all the data was anonymised, and the recordings deleted. Ethical approval was granted by the Research and Knowledge Exchange Ethics Committee at University of Winchester.

### Data analysis

Data was analysed between interviews, and interviews continued until codes found through analysis were ‘saturated’ [23]. Thus the process of data analysis was inductive with the themes derived from the data [24]. The software package NVivo supported this process by helping to manage the content from the interviews. The thematic approach saw codes initially independently identified by the authors (CC, AM, RL). The results of the coding were then compared and discussed by all the authors (CC, AM, RL, RML and MM) until there was convergence [25]. Coding was followed by the creation and development of themes - ‘interpretations of the issues under investigation’ [26]. Codes and themes are illustrated by quotations from interview participants (P). The rationale for the selection of quotations included in the results is they exemplified broader patterns in the data which were foregrounded through the analytic process.

## Results

The coded interviews were categorised into three broad and overlapping themes. These themes include working in an educationally minded culture; proximity of the educator to the trainee physician; and trajectory of the trainee physician. Table 1 lists these themes and includes selected quotes which further explain their meaning.

**Table 1:** Summary of findings

Themes	Actions	Methods	Quotes
**An educationally minded culture**	Regular discussion of physicians in training	Discuss physicians in training at departmental meetings. Have other frequent discussions between senior educators in the team.	‘Once a week, at our consultant or our senior meeting, we do a formal, we call it a trainee run-through. We discuss each trainee in turn...’ (P3)
	All team members are responsible for education	Listen to feedback about physicians in training from other members of the multidisciplinary team, for example senior nurses. Have an expectation that these team members can contribute to physicians in trainings’ education.	‘…we’ve got nurse practitioners so they get some information and guidance from them.’ (P5) ‘…there’s always actually quite a lot of people more senior around, not only doctors but nurses as well.’ (P10)
	Patient safety	Education and patient safety are seen as co-dependent. Physicians in training can be supported for longer period if required.	‘…we are aware that we have to be good sessional supervisors for the patient’s safety.’ (P4)
**Proximity of educator to physicians in training**	Working side by side	Working within the same space as the physician in training. Frequent formal and non-formal interactions fostering a rapport and sense of collegiality. Safe learning environment. First name terms.	‘…often we’ll be working by their side, looking after the patient as a team and we’ll be learning off each other.’ (P1) ‘Very much it’s first names to the physicians in training and consultants.’ (P2) ‘It’s almost like family.’ (P3)
	Observation	Allowing the physician in training to witness clerking and interactions between educator and patient.	‘I will role-model…so that the next time they might have done that process before they come and speak to me.’ (P11)
	Verbalisation of educator thought processes	Thinking about decision making aloud. Educator being open about their own fallibility and often themselves needing to seek further advice or information.	‘…do it aloud [decision making], weighing up the pros and cons…you’re teaching the junior doctor…’ (P12)
	Verbalisation of the physician in trainings’ thought processes	Ask physicians in training to verbalise their thought processes. Ask for the physician’s in training opinion.	‘…when they come and speak to me about a patient, I ask them to verbalise what their thinking is and then, [ask] okay, so what could it be if it’s not that?’ (P9)
**Trajectory of the physicians in training**	The beginning/Induction	Well-structured induction. Physician in training meets all team members. Role, responsibilities and expectations clear to physician in training. Close supervisor contact during this period.	‘during induction we ensure they understood…what’s expected of them in their role, how to access senior help, in hours and out of hours, who to go if they’re having difficulties, in or out of work…there’s several layers to the induction.’ (P4)
	Further On/Learning Arc	Multiple informal assessments. Allow more autonomy as physician in training gains experience and confidence. Allow more autonomy as you start to trust the physician in training. Accept mistakes will happen.	‘Gradually we encourage them, we sort of say, ‘No, you’re getting it right’…you can [have] more autonomy in the decision-making process…’ (P11)
	Individually Tailored Experience	Allow physicians in training more freedom in areas of experience. Give physicians in training support in areas of weakness. Maintain close supervision for longer if necessary.	‘…for the trainee who’s struggling, making sure they’re a bit better supported….that’s the trainee you keep on your ward round…you let one of the other trainee go off and be a bit more independent.’ (P13)

### Educationally minded culture

The departments in which these educators worked put a great importance on education of trainee physicians. All members of the team were responsible for education of the team, and all members, particularly senior nurses, were able to give feedback upon the trainee physicians’ progress:” We have that relationship with the senior nurses, that they will bring up any concerns. We’ll say, ‘How’s Sharon getting on? She was having a few difficulties a few weeks ago, is that getting any better?’, and they will give very honest feedback; that’s so helpful…” (P5). Trainee physicians’ progress was discussed regularly at formal departmental meetings:” We discuss each trainee in turn. If the clinical supervisor for that trainee is there, they’d take that and do the feedback to the trainee… On a regular basis all the consultants, not just individual clinical supervisors, are just discussing how each trainee is doing” (P8). Trainee physicians were also discussed informally: “All the consultants, we’re all on one corridor, and all of us with open doors, and we do regularly discuss trainees” (P3). Educators described that education was not only for the benefit of the trainee but that it was also essential for patient safety: “That’s really important for education but most importantly, that’s important for patient safety” (P9).

### Proximity of educator to trainee physicians

Educators described working side by side with their trainee physician and frequently being in close proximity to them, which means that the educator was both easily accessible and spent more time with their trainee physicians. For example, several of the educators described being on ‘the shop floor’ in the emergency department, spending most of their time working alongside their trainee physicians: “On the shop floor, you [the trainee physicians] can ask a consultant for advice...the most senior doctor” (P2). This closeness also helps foster a sense of collegiality, the trainee physicians being viewed as a colleague, through repeated formal and non-formal interactions, building rapport and a safe learning environment: “I think it’s having that flat hierarchy, having that community collegiality that helps make things easier” (P5).

This physical closeness also allowed the trainee physician to regularly witness the educator at work, meaning the trainee physician can model their own actions on that of their educator. The educators also described verbalising their thought processes in front of the trainee physician, giving clarity to the educators’ thought processes: “So they see us doing that [collecting the information from patients on admission] and modelling a way of working” (P12). As part of this, educators described being open and honest with trainee physicians about gaps in their own knowledge and how they dealt with that situation: If there is a problem as I don’t know what to do with X or Y, I say, “Look, this is quite clearly a haematological problem. I really don’t quite know what we should do with this. I know that the consultant haematologist is doing a ward round downstairs, let’s see them” (P9). Trainee physicians are also regularly encouraged to verbalise their thought processes: “What’s much better is to be able to see - almost see their thought process because you are watching them, seeing, talking to a patient, examining them, coming out, talking to nurses, ordering investigations, sitting, thinking, Googling headache or whatever” (P7).

### Trajectory of the trainee physicians

Many of the educators placed great importance on the process of induction at the start of the trainee physician’s placement in the department. The induction should be well organised and should allow the trainee physicians to meet the rest of the team and understand their role within the team: “They have an induction programme, so they know before they arrive who they’re going to be with and when….That induction typically goes on for about two-and-a-half weeks, so they can get a feel of all the different people at the surgery, what their different roles are, and get to know people a little bit better and how the surgery works before they’re thrown in at the deep end. During that induction, they may see patients, but very supervised…” (P4). This induction period also allows the educator to make multiple informal assessments regarding the trainee physician’s strengths and weaknesses and make a judgement regarding how much support they will require in different circumstances.

Educators described the trajectory of the trainee physicians through the placement with close monitoring and informal assessment throughout. Based on these observations and assessments they are gradually allowed to develop independence in their practice. Many educators described the process of building trust in the trainee physicians and allowing them to work more autonomously: “I can trust them. When they’re coming to me and they’re talking about my patients, I have a feeling of comfort and I’m not nervous” (P14).

As part of the induction, and the trainee physician’s development during the placement, educators described tailoring their placement for the trainee physicians, identifying their abilities in different areas and adjusting the clinical experience accordingly. In some placements trainee physicians were more closely supervised for longer periods if it was felt that not doing so would risk them being in a situation where they were out of their depth, even though this may impact on the service capacity of the department: “We say ‘We’d like you to continue after two weeks talking to a consultant for every patient’...for some trainees, because we don’t have consultants overnight, we actually stop them working after midnight” (P10).

Built into this progression during their placement was the sense that the educator was able to begin to trust the trainee physicians, allowing them to take more responsibility whilst at times letting them make mistakes: “Even if I don’t necessarily agree with it, as long as I’m happy that it’s safe, I’ll say, ‘Okay, let’s try that, and I’ll see the patient tomorrow, and we’ll see if it’s worked, see if it’s helping” (P11).

## Discussion

This study has sought to highlight the techniques and behaviours of educators and departments in providing the optimal context for trainee physicians to develop their clinical thinking abilities. The study reveals several ways educators and departments create an educationally minded culture, work in close proximity to trainee physicians and also allow trainee physicians to progress along a trajectory during their placement, to foster the development of clinical thinking. These themes have been identified, and a general framework of actions has been provided with specific examples which may optimise the development of clinical thinking (Table 1). These findings make two contributions. First, they extend the current medical education literature by adding the educator perspective on context and supportive learning environments, and are likely to be applicable to other areas of professional education. Second, the qualitative research approach we adopted here identifies and offers a narrative concerning that environment, revealing its importance for the development of medical practice in junior doctors, and, significantly, showing ways of discussing it. We believe that it is through encouraging and enabling this narrative to happen naturally as part of clinical teaching that change occurs.

The potential means by which educators and departments can introduce changes within their own practice and working environments have been compiled into a table of recommendations (Table 2). Educators may wish to select applicable recommendations for their own circumstances in order to make change. Modifications in workplace culture and working practices are most likely to be successful if locally driven, as opposed to being imposed by an external organisation.

**Table 2:** Recommendations for Educators and Departments

**Recommendations**	
**Educators**	
•Be prepared for induction period to be labour intensive	
•Be accessible and make sure trainees know how to contact you each day	
•Display fallibility	
•Exploit opportunities to work side by side with trainee	
•Verbalise thought processes	
•Tailor trainee experience throughout a placement	
•Be aware of trainees’ development through informal assessment of progress	
**Departments**	
•Offer structured and ongoing induction	
•Organise rotas so that the same staff work together and get to know the trainee	
•Place consultant educators’ offices close to one another	
•Prioritise education - all staff have a responsibility for education, with trainees discussed regularly and informally	
•Have a flat hierarchy	
•Regularly discuss at departmental meetings trainees’ progress and identify those in difficulty	

Well regarded departments were identified by feedback provided by trainee physicians; however not all educators approached agreed to participate. This is acknowledged by the authors as a limitation of the study as those that did not take part may have given different accounts to those that are represented here.

## Conclusion

Based on our findings there are several areas which could benefit from further research. To our knowledge this is the first study to examine this topic from the educator’s perspective and further studies with analysis across specialities could help to develop our findings. Several themes have been identified as enhancing the development of clinical thinking in the study; what has not been shown is how these could best be applied to help departments where these factors are not occurring. The findings suggest that allied health professionals played a role in assisting trainee physicians develop clinical thinking skills, and it would be of interest to study the interplay of how different healthcare professionals assist each other’s development of clinical thinking, which more accurately represents the multi-professional healthcare working environment. There is a lack of evidence linking positive fostering of development of clinical thinking and patient safety and patient outcomes. If such a link was shown this would give further weight to the importance of this topic.

## Data Availability

Data sharing not applicable to this article as no datasets were generated or analysed during the current study.

## Abbreviations

BERA: British Education Research Association
P: Participant
